# *Tournefortia sarmentosa* Inhibits the Hydrogen Peroxide-Induced Death of H9c2 Cardiomyocytes

**DOI:** 10.1155/2021/8219141

**Published:** 2021-08-26

**Authors:** Chih-Jen Liu, Lu-Kai Wang, Chan-Yen Kuo, Mao-Liang Chen, I-Shiang Tzeng, Fu-Ming Tsai

**Affiliations:** ^1^Division of Cardiology, Department of Internal Medicine, Taipei Tzu Chi Hospital, The Buddhist Tzu Chi Medical Foundation, New Taipei City 231, Taiwan; ^2^Radiation Biology Core Laboratory, Institute for Radiological Research, Chang Gung University/Chang Gung Memorial Hospital, Linkou, Taoyuan 333, Taiwan; ^3^Department of Research, Taipei Tzu Chi Hospital, The Buddhist Tzu Chi Medical Foundation, New Taipei City 231, Taiwan

## Abstract

*Tournefortia sarmentosa* is a traditional Chinese medicine used to reduce tissue swelling, to exert the antioxidant effect, and to detoxify tissue. *T. sarmentosa* is also used to promote development in children and treat heart dysfunction. However, many of the mechanisms underlying the effects of *T. sarmentosa* in the treatment of disease remain unexplored. In this study, we investigated the antioxidant effect of *T. sarmentosa* on rat H9c2 cardiomyocytes treated with hydrogen peroxide (H_2_O_2_). *T. sarmentosa* reduced the cell death induced by H_2_O_2_. *T. sarmentosa* inhibited H_2_O_2_-induced changes in cell morphology, activation of cell death-related caspases, and production of reactive oxygen species. In addition, we further analyzed the potential active components of *T. sarmentosa* and found that the compounds present in the *T. sarmentosa* extract, including caffeic acid, rosmarinic acid, salvianolic acid A, and salvianolic acid B, exert effects similar to those of the *T. sarmentosa* extract in inhibiting H_2_O_2_-induced H9c2 cell death. Therefore, according to the results of this study, the ability of the *T. sarmentosa* extract to treat heart disease may be related to its antioxidant activity and its ability to reduce the cellular damage caused by free radicals.

## 1. Introduction

Oxidative stress caused by free radicals is associated with many cardiovascular diseases, including ischemic heart disease, atherosclerosis, hypertension, and cardiomyopathies [1–4]. Free radicals are generated in the human body during the process of energy production. When the body suffers from inflammation, such as inflammation elicited by infection or injury, many free radicals are produced to remove foreign substances [5]. However, if the host does not have a sufficient protective mechanism to remove excess free radicals after infection, these free radicals will cause cell damage (including lipid peroxidation of the cell membrane and DNA damage) [6, 7]. In addition, environmental factors such as ultraviolet radiation, chemical drugs, and even excessive pressure result in a substantial increase in the production of free radicals in the body [8]. When cardiac muscle cells are attacked by free radicals, the free radicals-induced injury can damage nuclear DNA and lead to permanent DNA damage. The lipids in the cell membrane can be oxidized, altering cell membrane fluidity, preventing nutrients from entering the cell, and eventually causing necrosis. Free radicals attack the side chains of amino acids, causing proteins to lose their function and disrupting the normal functions of cells [5].

Several antioxidant enzymes in the human body, such as superoxide dismutase, glutathione peroxidase, or catalase, counteract free radicals. These enzymes quickly convert the free radicals produced by the body into less toxic or nontoxic substances through redox reactions [9, 10]. However, when the activity of such antioxidant enzymes is insufficient due to insufficient intake of cofactors (superoxide dismutase requires metal ions, such as Cu^2+^ or Zn^2+^) or differences in genetic background, cells are unable to avoid free radicals attack and subsequent damage [11]. When a tissue is damaged and dies, it can be restored by the proliferation of stem cells present in the tissue in order to maintain the normal operation of the organ or tissue. However, the existence of cardiac stem cells remains controversial [12]. Therefore, methods to prevent cardiomyocyte damage by free radicals or to reduce cells damage caused by the oxidative pressure generated by free radicals are important topics at present.

*Tournefortia sarmentosa* is often mentioned in reports that evaluate its antioxidant activity in Chinese medicine [13, 14]. *T. sarmentosa* also exerts the effects of relieving wind, detoxifying, reducing swelling, treating aching muscles and bones, and promoting childhood development [15–17]. In addition to being used as a specific therapeutic drug in traditional Chinese medicine, *T. sarmentosa* has recently been promoted as a health food. However, although several studies have investigated the antioxidant effect of *T. sarmentosa*, researchers have not determined whether *T. sarmentosa* protects cardiomyocytes from oxidative stress. In this study, we investigated the protective effect of *T. sarmentosa* on hydrogen peroxide- (H_2_O_2_-) induced myocardial cell death. In addition to the protective effect of *T. sarmentosa* on cardiomyocytes, we also analyzed the potential active components of *T. sarmentosa* that protect against cardiomyocyte death.

## 2. Materials and Methods

### 2.1. Preparation of an Aqueous Extract of *T. sarmentosa* and Reagents

The preparation of the aqueous extract of *T. sarmentosa* was described in a previous study [14]. The various components of the *T. sarmentosa* extract, including caffeic acid, rosmarinic acid, salvianolic acid A, and salvianolic acid B, were purchased from Sigma-Aldrich (St. Louis, MO, USA).

### 2.2. Cell Culture

Rat H9c2 (2-1) cardiomyocytes were purchased from Bioresource Collection and Research Center (Hsinchu city, Taiwan). The cells were cultured in 90% Dulbecco's modified Eagle's medium (DMEM) containing 4 mM L-glutamine, 1.5 g/L sodium bicarbonate, 4.5 g/L glucose, and 10% fetal bovine serum at 37°C in a 5% carbon dioxide environment. During cell culture, mycoplasma contamination was regularly evaluated, and mycoplasma contamination was not detected.

### 2.3. Detection of Cell Viability and Cell Death

WST-1 reagent (Roche Diagnostics, Mannheim, Germany) and a cytotoxicity detection kit (Roche Diagnostics) were used to measure cell viability and cell death, respectively [18]. H9c2 cells were plated in a 6-well plate in triplicate at a density of 10^5^ cells/well and cultured overnight. The medium was replaced with serum-free DMEM, and various doses of *T. sarmentosa* or 100 *μ*m solutions of the components present in *T. sarmentosa* were added and incubated for another 24 h. After incubation with WST-1 for 3 h and 100 *μ*M H_2_O_2_ for another 1 h, the supernatants were collected, and the absorbance values were measured at 450 nm and 650 nm using a multifunctional microplate instrument (Infinite F200, Tecan, Durham, NC, USA). Alternatively, cells growing in the serum-free medium were cultured with various concentrations of *T. sarmentosa* or 100 *μ*m solutions of the components present in *T. sarmentosa* for 24 h, followed by incubation with 100 *μ*M H_2_O_2_ for 1 h. The samples were then centrifuged, and the supernatant of each sample was collected. The amounts of lactate dehydrogenase present in the supernatants were detected with a cytotoxicity detection kit.

### 2.4. Western Blotting

H9c2 cells were plated in a 10 cm dish at a density of 10^6^ cells/dish and cultured overnight. After the cells were cultured in the serum-free medium supplemented with various doses of *T. sarmentosa* or 100 *μ*m components present in *T. sarmentosa* for 24 h, the cells were treated with 100 *μ*M H_2_O_2_ for 1 h, and the cell extracts were collected with mammalian protein extraction buffer (GE Healthcare Bio-Sciences, MJ, USA). The expression of protein in each sample was analyzed using Western blotting, as described in the previous literature [19]. Caspase-8, caspase-9, and caspase-3 antibodies were purchased from Cell Signaling Technology Inc. (Beverly, MA, USA). The actin antibody was purchased from Sigma-Aldrich.

### 2.5. Reactive Oxygen Species (ROS) Measurement

Dihydrorhodamine 123 (DHR123) dye was used to detect ROS levels in cells as previously described [14]. Briefly, H9c2 cells were plated in a 10 cm dish at a density of 10^6^ cells/dish and cultured overnight. After the cells were cultured in serum-free medium supplemented with various doses of *T. sarmentosa* or 100 *μ*m components present in *T. sarmentosa* for 24 h, the cells were treated with 100 *μ*M H_2_O_2_ and 2.5 *μ*M DHR123 for 1 h. The cells were then trypsinized and passed through a 30 *μ*m filter, and the ROS contents in the cells were analyzed with a flow cytometer (FACScan, Becton Dickinson, Franklin Lakes, NJ, USA).

### 2.6. Statistical Analysis

The experimental data are presented as the average value ± SD of triplicate samples from each independent biological sample. Statistical analysis was analyzed using Student's *t*-test, and *p* values less than 0.05 were considered significant.

## 3. Results

### 3.1. *T. sarmentosa* Prevented the H_2_O_2_-Induced Death of H9c2 Cardiomyocytes

**Figure 1:**
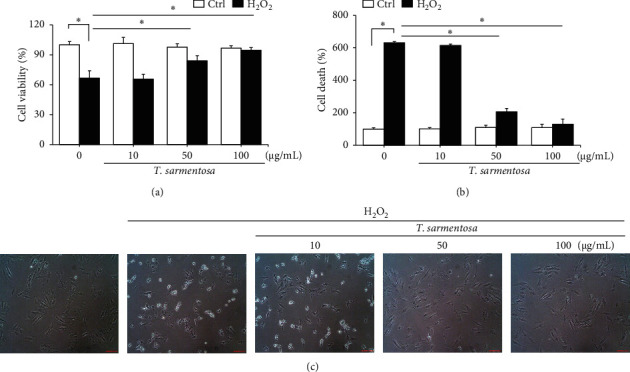
*T. sarmentosa* extract inhibiting H_2_O_2_-induced H9c2 cell death. H9c2 cells are treated with different doses of the *T. sarmentosa* extract for 24 h and then treated with 100 *μ*M H_2_O_2_ for 1 h. Cell viability (a) and cell death (b) are detected using WST-1 reagent or by measuring the amount of LDH release, respectively. Cell morphology observed under a light microscope at 40x magnification (c). ^*∗*^*P* < 0.05 for comparisons between two groups.

H9c2 cells were selected as the experimental material and treated with 100 *μ*M H_2_O_2_ for one hour to induce cell death as described in a previously published study in order to determine whether ROS caused cardiomyocyte death [20]. As shown in Figures 1(a) and 1(b), H_2_O_2_ significantly reduced the cell survival rate and increased the number of dead H9c2 cells. However, when the cells were treated with increasing doses of *T. sarmentosa*, the H_2_O_2_-induced decrease in the cell survival rate and increase in the cell death rate were significantly inhibited (Figures 1(a) and 1(b)). When the cells were treated with different doses of *T. sarmentosa* alone, no significant changes in cell survival or cell death were observed, suggesting that *T. sarmentosa* is not cytotoxic to H9c2 cells. In addition, the morphology of the cells was observed directly under a microscope. When the cells were treated with H_2_O_2_, the spindle-like morphology of the cells was not maintained, and the cells detached. When the cells were pretreated with *T. sarmentosa*, especially at a high dose of 50–100 *μ*g/mL, the cell morphology recovered noticeably. Based on these results, *T. sarmentosa* inhibited the changes in cell morphology and cell death induced by H_2_O_2_ (Figure 1(c)).

### 3.2. *T. sarmentosa* Reduced the Levels of Apoptosis-Related Caspases and Increased the Production of ROS Induced by H_2_O_2_

**Figure 2:**
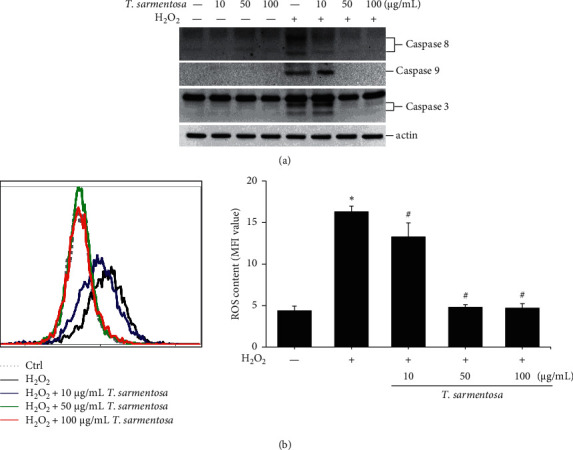
*T. sarmentosa* extract inhibiting the H_2_O_2_-induced expression of cell death-related caspases and production of ROS. H9c2 cells are treated with different doses of the *T. sarmentosa* extract for 24 h and then treated with 100 *μ*M H_2_O_2_ for 1 h. The levels of cell death-related caspases are analyzed using Western blotting (a). ROS production is detected by staining the cells with the DHR123 dye and analyzed using flow cytometry (b).

Since *T. sarmentosa* inhibited the H_2_O_2_-induced death of H9c2 cells, we further explored whether *T. sarmentosa* altered the H_2_O_2_-induced activation of cell death-related signal transduction pathways. In the cells treated with different doses of *T. sarmentosa*, no caspase-8, caspase-9, or caspase-3 expression was detected. However, when the cells were treated with H_2_O_2_, the levels of caspase-8, caspase-9, and caspase-3 significantly increased. When the cells were pretreated with high doses of 50–100 *μ*g/mL *T. sarmentosa*, the H_2_O_2_-induced increases in the levels of the death-related caspases mentioned above were significantly reduced (Figure 2(a)).

Cell death caused by H_2_O_2_ is closely related to ROS production. Therefore, we further verified whether the inhibition of H_2_O_2_-induced cell death by *T. sarmentosa* is related to ROS production. As shown in Figure 2(b), H_2_O_2_ significantly increased the intracellular ROS levels, and the H_2_O_2_-induced increase in the intracellular ROS content was significantly decreased by treatment with increasing doses of *T. sarmentosa*.

### 3.3. All the Compounds Present in the *T. sarmentosa* Extract Inhibited H_2_O_2_-Induced H9c2 Cell Death

**Figure 3:**
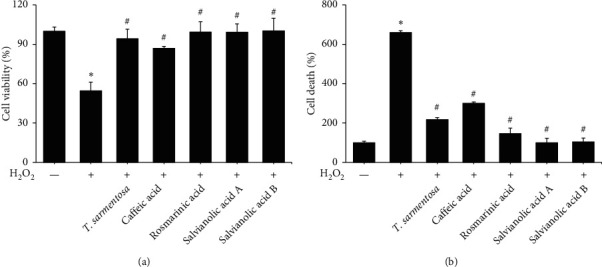
The compounds present in the *T. sarmentosa* extract inhibiting H_2_O_2_-induced H9c2 cell death. H9c2 cells are treated with 100 *μ*m solutions of the indicated compounds present in the *T. sarmentosa* extract for 24 h and then treated with 100 *μ*M H_2_O_2_ for 1 h. Cell viability (a) and cell death (b) are detected using the WST-1 reagent or by measuring the amount of LDH release, respectively. ^*∗*^*P* < 0.05 compared with the control group. ^#^*P* < 0.05 compared with the control group treated with H_2_O_2_.

We selected several compounds present in the *T. sarmentosa* extract that are commercially available to further explore the components of *T. sarmentosa* that effectively inhibit H_2_O_2_-induced H9c2 cell death [21]. As shown in Figures 3(a) and 3(b), caffeic acid, rosmarinic acid, salvianolic acid A, and salvianolic acid B exhibited effects similar to those of *T. sarmentosa*, as they significantly reversed the H_2_O_2_-induced decrease in the H9c2 cell survival rate and increase in the cell death rate.

### 3.4. All Compounds Present in the *T. sarmentosa* Extract Decreased the H_2_O_2_-Induced Increases in the Levels of Apoptosis-Related Caspases and Production of ROS

**Figure 4:**
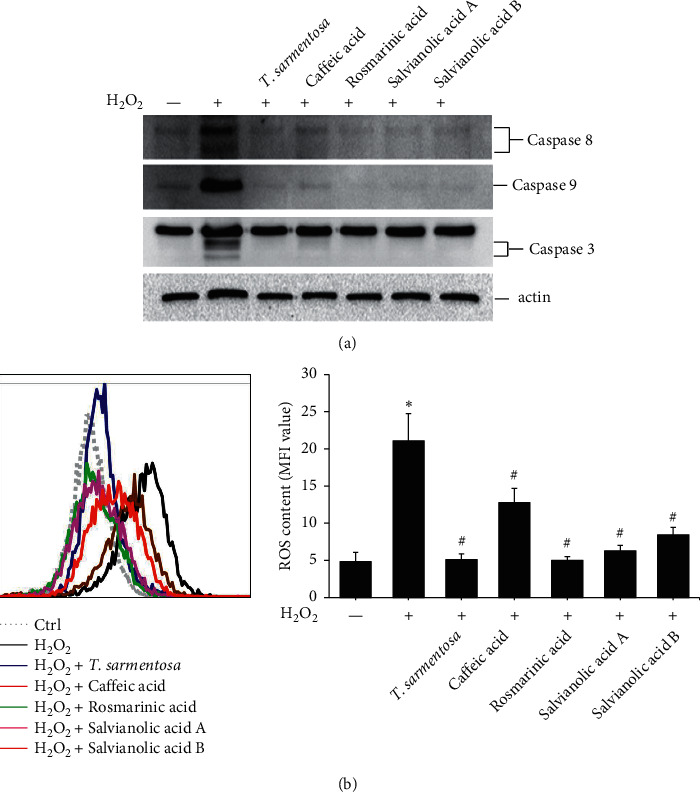
*T. sarmentosa* extract inhibiting the H_2_O_2_-induced expression of cell death-related caspases and production of ROS. H9c2 cells treated with 100 *μ*M solutions of the indicated compounds present in the *T. sarmentosa* extract for 24 h and then treated with 100 *μ*M H_2_O_2_ for 1 h; the level of cell death-related caspases is analyzed by Western blotting (a). The production of ROS is measured with the DHR123 dye and analyzed by flow cytometry (b). ^*∗*^*P* < 0.05 compared with the control group. ^#^*P* < 0.05 compared with the control group treated with H_2_O_2_.

We further analyzed whether the compounds present in the *T. sarmentosa* extract also altered the H_2_O_2_-induced expression of caspases and production of ROS. H_2_O_2_ induced the expression of caspases, and except for caffeic acid, which had only a slight effect on the expression of caspase-3, the H_2_O_2_-induced expression of caspase-8, caspase-9, and caspase-3 was inhibited by all the compounds found in *T. sarmentosa* extract (Figure 4(a)). In addition, we also analyzed the effects of these compounds on the H_2_O_2_-induced intracellular ROS production. As shown in Figure 4(b), all the compounds present in the *T. sarmentosa* extract significantly inhibited the H_2_O_2_-induced ROS production.

## 4. Discussion

*T. sarmentosa* is widely distributed and is found in Taiwan, Vietnam, Malaysia, the Philippines, Indonesia, and many other regions. *T. sarmentosa* is also widely used as a medicinal material. For example, the stems and leaves are mashed and taken orally with wine to promote blood circulation and treat old and new injuries. The medical efficacy of this plant extract has consistently been reported; however, few studies have explored its potential efficacy because the use of traditional Chinese medicine is not often investigated in many scientific studies.

In this study, we confirmed the antioxidant effect of *T. sarmentosa* on cardiomyocytes, but it only inhibited H_2_O_2_-induced cell death at higher doses. Although *T. sarmentosa* extract has been used as a traditional Chinese medicine in humans for years, no studies have reported its concentration in human blood after consumption; thus, researchers have not determined whether the dose exerts a cardioprotective antioxidant effect. However, *T. sarmentosa* was orally administered at doses of 2,000 mg/kg bodyweight in in vivo studies and at doses of 10–100 mg/mL in cell-based experiments [15]. Therefore, the concentration of the *T. sarmentosa* extract tested in this study should be within a reasonable range. Magnolol is the most popular Chinese medicine used to protect the myocardium and possesses antioxidant activity. The oral dose of *Magnolia officinalis* extract in live animals ranges from 0.48 to 2.5 g/kg/day [22, 23], which was not significantly different from the *T. sarmentosa* dose. In addition, the half-life of magnolol in vivo and the organs to which the drug is distributed after injection have been reported in a review [24]. Therefore, compared with magnolol, the cardioprotective effects of *T. sarmentosa* still need to be verified in additional experiments.

The efficacy of *T. sarmentosa* is related to its individual components. The individual compounds present in the *T. sarmentosa* extract used in this study, including caffeic acid, rosmarinic acid, and salvianolic acid, have a benzene ring structure, which has antioxidant properties [25]. In addition, the various compounds present in *T. sarmentosa* mentioned above have been reported to possess antioxidant properties [26–32]. Although different plants exert effects similar to those of traditional Chinese medicines, many differences in their internal components have been noted, and these differences also result in their different medicinal purposes. For example, many components of *T. sarmentosa* are similar to those of *Salvia officinalis* [33], but significant differences were observed after a further analysis of the components [34]; thus, the two plants have different therapeutic purposes. *Salvia officinalis* is mainly used for relieving anxiety, while *T. sarmentosa* is mainly used for detoxification and to exert the antioxidant effect.

Many studies have reviewed the antioxidant, anti-inflammatory, and antiangiogenic properties of caffeic acid that exert important antiatherosclerotic effects and protect tissues from the ischemia/reperfusion injury and cell dysfunction caused by different physical and chemical agents [35, 36]. Rosmarinic acid has been shown to protect against inflammation and myocardial cell apoptosis during myocardial ischemia/reperfusion injury by activating peroxisome proliferator-activated receptor *γ* and inhibiting the production of inflammatory cytokines, such as IL-6, TNF-*α*, and C-reactive protein [37]. In addition, rosmarinic acid has been shown to improve cardiac dysfunction and mitochondrial damage in mice with diabetic cardiomyopathy by activating the SIRT1/PGC-1*α* signaling pathway [38]. The pathway by which salvianolic acid A exerts its protective effects was similar to that of rosmarinic acid. Arsenic trioxide induced the production of ROS and decreased the expression level of PGC-1*α* in cardiomyocytes. The addition of salvianolic acid A could restore the above phenomena to reduce cardiac mitochondrial damage and improve the cardiotoxicity induced by arsenic trioxide [39]. The protective potential of compounds found in the *T. sarmentosa* extract is closely related to the benzene ring structure of these compounds.

In addition to traditional Chinese medicine, coenzyme Q10 (CoQ10) is widely used in the market as a health food. CoQ10 is a coenzyme that is indispensable in the human body. CoQ10 exists in cells and is mainly distributed in the heart, kidneys, liver, and muscles. CoQ10 functions to stimulate mitochondrial energy production. In addition, CoQ10 functions as an antioxidant in the mitochondria, is involved in the energy production process, and reduces free radicals. Damage to mitochondria maintains the integrity and stability of cells, slows the oxidation of bad cholesterol, delays aging, and activates the immune system [40]. The effective dose of CoQ10 for the prevention of cardiovascular disease is approximately 100–300 mg [41–43]. However, due to the regulatory limitations of many local laws and regulations, high doses of CoQ10 are not available to many people, and thus, its efficacy is controversial. From the perspective of the structures of the compounds, CoQ10 and *T. sarmentosa* extracts contain similar antioxidant components that have benzene ring structures and unsaturated hydrocarbon chains, indicating the potential use of *T. sarmentosa* as a health food for heart protection.

## 5. Conclusions

In summary, the *T. sarmentosa* extract and several compounds present in the *T. sarmentosa* extract inhibit the H_2_O_2_-induced death of H9c2 cardiomyocytes. Furthermore, the *T. sarmentosa* extract and several of its compounds inhibit the H_2_O_2_-induced expression of cell death-related caspases and production of ROS. Therefore, the *T. sarmentosa* extract may have potential for use as a cardioprotective health food.

## Data Availability

The data used to support the results of this study are included within the article.

## References

[1] D’Oria R., Schipani R., Leonardini A. (2020). The role of oxidative stress in cardiac disease: from physiological response to injury factor. *Oxidative Medicine and Cellular Longevity*.

[2] Campos R. R. (2009). Oxidative stress in the brain and arterial hypertension. *Hypertension Research*.

[3] Romero-Alvira D., Roche E., Placer L. (1996). Cardiomyopathies and oxidative stress. *Medical Hypotheses*.

[4] Dhalla N. S., Temsah R. M., Netticadan T. (2000). Role of oxidative stress in cardiovascular diseases. *Journal of Hypertension*.

[5] Cadenas E., Davies K. J. (2000). Mitochondrial free radical generation, oxidative stress, and aging. *Free Radical Biology & Medicine*.

[6] Lagouge M., Larsson N. G. (2013). The role of mitochondrial DNA mutations and free radicals in disease and ageing. *Journal of Internal Medicine*.

[7] Warraich U.-E.-A., Hussain F., Kayani H. U. R. (2020). Aging—oxidative stress, antioxidants and computational modeling. *Heliyon*.

[8] Phaniendra A., Jestadi D. B., Periyasamy L. (2015). Free radicals: properties, sources, targets, and their implication in various diseases. *Indian Journal of Clinical Biochemistry*.

[9] Pigeolet E., Corbisier P., Houbion A. (1990). Glutathione peroxidase, superoxide dismutase, and catalase inactivation by peroxides and oxygen derived free radicals. *Mechanism of Ageing and Development*.

[10] Aruoma O. I., Halliwell B. (1987). Action of hypochlorous acid on the antioxidant protective enzymes superoxide dismutase, catalase and glutathione peroxidase. *Biochemical Journal*.

[11] Mondola P., Damiano S., Sasso A., Santillo M. (2016). The Cu, Zn superoxide dismutase: not only a dismutase enzyme. *Frontiers in Physiology*.

[12] Kretzschmar K., Post Y., Bannier-Hélaouët M. (2018). Profiling proliferative cells and their progeny in damaged murine hearts. *Proceedings of the National Academy of Sciences*.

[13] Lin Y.-L., Chang Y.-Y., Kuo Y.-H., Shiao M.-S. (2002). Anti-lipid-peroxidative principles from Tournefortia sarmentosa. *Journal of Natural Products*.

[14] Chen M.-L., Wu S., Tsai T.-C., Wang L.-K., Chou W.-M., Tsai F.-M. (2013). Effect of aqueous extract of Tournefortia sarmentosa on the regulation of macrophage immune response. *International Immunopharmacology*.

[15] Teng C.-Y., Lai Y.-L., Huang H.-I., Hsu W.-H., Yang C.-C., Kuo W.-H. (2012). Tournefortia sarmentosaextract attenuates acetaminophen-induced hepatotoxicity. *Pharmaceutical Biology*.

[16] Wang L. K., Tsai F. M., Wu S. (2018). Aqueous extract of tournefortia sarmentosa stem inhibits ADP-induced platelet aggregation. *Indian Journal of Pharmaceutical Sciences*.

[17] Lans C. (2007). Comparison of plants used for skin and stomach problems in trinidad and tobago with Asian ethnomedicine. *Journal of Ethnobiology and Ethnomedicine*.

[18] Wu C.-C., Shyu R.-Y., Wang C.-H. (2012). Involvement of the prostaglandin D2 signal pathway in retinoid-inducible gene 1 (RIG1)-mediated suppression of cell invasion in testis cancer cells. *Biochimica et Biophysica Acta (BBA)—Molecular Cell Research*.

[19] Wang C. C., Wang L. K., Chen M. L., Kuo C. Y., Tsai F. M., Wang C. H. (2020). Triterpenes in the ethanol extract of poria cocos induce dermal papilla cell proliferation. *International Journal of Pharmacology*.

[20] Chang H., Li C., Huo K. (2016). Luteolin prevents H_2_O_2_-induced apoptosis in H9C2 cells through modulating Akt-P53/mdm2 signaling pathway. *BioMed Research International*.

[21] Lin Y.-L., Tsai Y.-L., Kuo Y.-H., Liu Y.-H., Shiao M.-S. (1999). Phenolic compounds from tournefortia sarmentosa. *Journal of Natural Products*.

[22] Li N., Song Y., Zhang W. (2007). Evaluation of the in vitro and in vivo genotoxicity of magnolia bark extract. *Regulatory Toxicology and Pharmacology*.

[23] Liu Z., Zhang X., Cui W. (2007). Evaluation of short-term and subchronic toxicity of magnolia bark extract in rats. *Regulatory Toxicology and Pharmacology*.

[24] Ho J., Hong C.-Y. (2012). Cardiovascular protection of magnolol: cell-type specificity and dose-related effects. *Journal of Biomedical Science*.

[25] Chen J., Yang J., Ma L., Li J., Shahzad N., Kim C. K. (2020). Structure-antioxidant activity relationship of methoxy, phenolic hydroxyl, and carboxylic acid groups of phenolic acids. *Scientific Reports*.

[26] Espíndola K. M. M., Ferreira R. G., Narvaez L. E. M. (2019). Chemical and pharmacological aspects of caffeic acid and its activity in hepatocarcinoma. *Frontiers in Oncology*.

[27] Gülçin I. (2006). Antioxidant activity of caffeic acid (3,4-dihydroxycinnamic acid). *Toxicology*.

[28] Genaro-Mattos T. C., Maurício Â. Q., Rettori D., Alonso A., Hermes-Lima M. (2015). Antioxidant activity of caffeic acid against iron-induced free radical generation-a chemical approach. *PLoS One*.

[29] Adomako-Bonsu A. G., Chan S. L., Pratten M., Fry J. R. (2017). Antioxidant activity of rosmarinic acid and its principal metabolites in chemical and cellular systems: importance of physico-chemical characteristics. *Toxicology in Vitro*.

[30] Taguchi R., Hatayama K., Takahashi T. (2017). Structure-activity relations of rosmarinic acid derivatives for the amyloid *β* aggregation inhibition and antioxidant properties. *European Journal of Medicinal Chemistry*.

[31] Xu H., Li Y., Che X., Tian H., Fan H., Liu K. (2014). Metabolism of salvianolic acid A and antioxidant activities of its methylated metabolites. *Drug Metabolism and Disposition*.

[32] Ma L., Tang L., Yi Q. (2019). Salvianolic acids: potential source of natural drugs for the treatment of fibrosis disease and cancer. *Frontiers in Pharmacology*.

[33] Bors W., Michel C., Stettmaier K., Lu Y., Foo L. Y. (2004). Antioxidant mechanisms of polyphenolic caffeic acid oligomers, constituents of salvia officinalis. *Biological Research*.

[34] Uritu C. M., Mihai C. T., Stanciu G. D. (2018). Medicinal plants of the family lamiaceae in pain therapy: a review. *Pain Research & Management*.

[35] Silva H., Lopes N. M. F. (2020). Cardiovascular effects of caffeic acid and its derivatives: a comprehensive review. *Frontiers in Physiology*.

[36] Parlakpinar H., Sahna E., Acet A., Mizrak B., Polat A. (2005). Protective effect of caffeic acid phenethyl ester (CAPE) on myocardial ischemia-reperfusion-induced apoptotic cell death. *Toxicology*.

[37] Han J., Wang D., Ye L. (2017). Rosmarinic acid protects against inflammation and cardiomyocyte apoptosis during myocardial ischemia/reperfusion injury by activating peroxisome proliferator-activated receptor gamma. *Frontiers in Pharmacology*.

[38] Diao J., Zhao H., You P. (2021). Rosmarinic acid ameliorated cardiac dysfunction and mitochondrial injury in diabetic cardiomyopathy mice via activation of the SIRT1/PGC-1*α* pathway. *Biochemical and Biophysical Research Communications*.

[39] Zhang J.-Y., Wang M., Wang R.-Y. (2018). Salvianolic acid A ameliorates arsenic trioxide-induced cardiotoxicity through decreasing cardiac mitochondrial injury and promotes its anticancer activity. *Frontiers in Pharmacology*.

[40] Zozina V. I., Covantev S., Goroshko O. A., Krasnykh L. M., Kukes V. G. (2018). Coenzyme Q10 in cardiovascular and metabolic diseases: current state of the problem. *Current Cardiology Reviews*.

[41] Mortensen S. A., Rosenfeldt F., Kumar A. (2014). The effect of coenzyme Q10 on morbidity and mortality in chronic heart failure: results from Q-SYMBIO: a randomized double-blind trial. *Journal of the American College of Cardiology: Heart Failure*.

[42] Ghule A. E., Kulkarni C. P., Bodhankar S. L., Pandit V. A. (2009). Effect of pretreatment with coenzyme Q10 on isoproterenol-induced cardiotoxicity and cardiac hypertrophy in rats. *Current Therapeutic Research*.

[43] Lee B.-J., Tseng Y.-F., Yen C.-H., Lin P.-T. (2013). Effects of coenzyme Q10 supplementation (300 mg/day) on antioxidation and anti-inflammation in coronary artery disease patients during statins therapy: a randomized, placebo-controlled trial. *Nutrition Journal*.

